# Positional preferences of acetyl esterases from different CE families towards acetylated 4-*O*-methyl glucuronic acid-substituted xylo-oligosaccharides

**DOI:** 10.1186/s13068-014-0187-6

**Published:** 2015-01-22

**Authors:** Klaus G Neumüller, Adriana Carvalho de Souza, Jozef HJ van Rijn, Hugo Streekstra, Harry Gruppen, Henk A Schols

**Affiliations:** DSM Biotechnology Center, PO Box 1, 2600 MA Delft, The Netherlands; Laboratory of Food Chemistry, Wageningen University, Bornse Weilanden 9, 6708 WG Wageningen, The Netherlands

**Keywords:** Acetyl xylan esterase, Positional specificity, Xylan, Deacetylation, Hemicellulase

## Abstract

**Background:**

Acetylation of the xylan backbone restricts the hydrolysis of plant poly- and oligosaccharides by hemicellulolytic enzyme preparations to constituent monosaccharides. The positional preferences and deacetylation efficiencies of acetyl esterases from seven different carbohydrate esterase (CE) families towards different acetylated xylopyranosyl units (Xyl*p*) - as present in 4-*O*-methyl-glucuronic acid (MeGlcA)-substituted xylo-oligosaccharides (AcUXOS) derived from *Eucalyptus globulus* - were monitored by ^1^H NMR, using common conditions for biofuel production (pH 5.0, 50°C).

**Results:**

Differences were observed regarding the hydrolysis of 2-*O*, 3-*O*, and 2,3-di-*O* acetylated Xyl*p* and 3-*O* acetylated Xyl*p* 2-*O* substituted with MeGlcA. The acetyl esterases tested could be categorized in three groups having activities towards (i) 2-*O* and 3-*O* acetylated Xyl*p*, (ii) 2-*O*, 3-*O*, and 2,3-di-*O* acetylated Xyl*p*, and (iii) 2-*O*, 3-*O*, and 2,3-di-*O* acetylated Xyl*p*, as well as 3-*O* acetylated Xyl*p* 2-*O* substituted with MeGlcA at the non-reducing end. A high deacetylation efficiency of up to 83% was observed for CE5 and CE1 acetyl esterases. Positional preferences were observed towards 2,3-di-*O* acetylated Xyl*p* (*Te*CE1, *An*CE5, and *Os*CE6) or 3-*O* acetylated Xyl*p* (*Ct*CE4).

**Conclusions:**

Different positional preferences, deacetylation efficiencies, and initial deacetylation rates towards 2-*O*, 3-*O*, and 2,3-di-*O* acetylated Xyl*p* and 3-*O* acetylated Xyl*p* 2-*O* substituted with MeGlcA were demonstrated for acetyl esterases from different CE families at pH 5.0 and 50°C. The data allow the design of optimal, deacetylating hemicellulolytic enzyme mixtures for the hydrolysis of non-alkaline-pretreated bioenergy feedstocks.

**Electronic supplementary material:**

The online version of this article (doi:10.1186/s13068-014-0187-6) contains supplementary material, which is available to authorized users.

## Background

Xylan is a valuable source of C5 sugars for use in biorefineries. However, acetylation of xylan restricts enzymatic degradation of the xylan backbone [1]. Therefore, enzymatic hydrolysis of glycosidic substituents and phenyl and acetyl esters increases the accessibility of the (1 through 4)-β-D-xylan backbone for β-1,4-endo-xylanases and β-1,4-xylosidases. The Carbohydrate-Active enzymes (CAZy) database classification (www.cazy.org) recognizes 16 carbohydrate esterase (CE) families. Unfortunately, a lack of knowledge of the sequence-to-specificity relationships in CAZy families does not yet allow a reliable, automated substrate prediction [2] for the esterases within a CE family.

Carbohydrate esterases with activity towards acetylated xylan were reported to fall into eight different CE families (CE1, 2, 3, 4, 5, 6, 7, and 16) [3,4]. As reviewed previously [3], the positional preference on synthetic substrates has been described for some acetyl xylan esterases. A characterization of the positional preference of acetyl xylan esterases on partially acetylated xylo-oligosaccharides by NMR is only available for the CE1 (*Schizophyllum commune*), CE4 (*Streptomyces lividans*), and CE6 (*Orpinomyces* sp.) esterases [5,6]. In only a few cases has the specificity towards different acetylated xylopyranosyl units (Xyl*p*) also been investigated on actual plant oligosaccharides [7,8], and even in those cases the deacetylation rates towards the differently acetylated Xyl*p* were not reported. Knowledge of these rates is required to determine the positional preference of the acetyl esterases in the early stage of the reaction when many competing substrates are still present.

Using artificial substrates, it has been shown that CE1 and CE5 acetyl xylan esterases have a strong preference for the deacetylation of 2-*O* acetyl 4-nitrophenyl β-D-xylopyranoside [9]. However, esterases from these families were reported to also be active on 3-*O* acetylated xylopyranosyl residues if plant-derived xylo*-*oligosaccharides were used [7]. This shows that one cannot rely solely on artificial substrates to characterize the positional preference of acetyl esterases, and that natural substrates are required as well. Both the initial deacetylation rates and the final deacetylation efficiency on differently acetylated, plant-derived Xyl*p* residues are required to characterize the acetyl esterases from different CE families. Moreover, these measurements should preferably be performed under hydrolysis conditions relevant for the production of industrial biofuels, since it cannot be excluded that these conditions affect the positional preference.

Recently, it was shown that supplementation of a hemicellulolytic enzyme preparation containing CE1 acetyl xylan esterases with a CE5 acetyl xylan esterase from *Trichoderma longibrachiatum* increased substrate conversion to monomers [10]. Addition of acetyl xylan esterases to hemicellulolytic enzyme preparations allows efficient hydrolysis of hemicellulose when harsh alkaline pretreatments [11] are reduced. This shows the potential relevance of (the right mix of) acetyl esterases for efficient hemicellulose degradation.

Here, we describe the deacetylation by carbohydrate esterases from different CE families on acetylated 4-*O*-methyl glucuronic acid (MeGlcA)-substituted xylo-oligosaccharides (AcUXOS) at pH 5 and 50°C, which are common conditions for biofuel production [12,13] and conditions under which the acetyl esterases are active. ^1^H and 2D NMR spectra were used to characterize the deacetylation of 2-*O*, 3-*O*, and 2,3-di-*O* acetylated Xyl*p* and the 3-*O* acetylated Xyl*p* 2-*O* substituted with MeGlcA. The positional preference by the enzymes towards differently acetylated Xyl*p* is presented.

## Results and discussion

### Acetyl esterases

**Figure 1:**
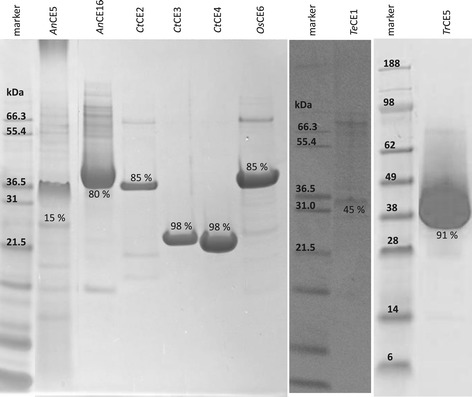
**SDS-PAGE acetyl esterases.** SDS-PAGE of acetyl esterases from *Aspergillus niger* (*An*CE5, *An*CE16), *Clostridium thermocellum* (*Ct*CE2, *Ct*CE3, *Ct*CE4), *Orpinomyces* sp*.* (*Os*CE6), *Talaromyces emersonii* (*Te*CE1), and *Trichoderma reesei* (*Tr*CE5). The purity of the acetyl esterases present is shown as densimetric proportion (%) of the total proteins present as determined by SDS-PAGE analysis. The protein markers Mark 12 and SeeBlue Plus 2 were used.

Selected acetyl esterases were analyzed for their purity by SDS-PAGE (Figure 1). The protein bands of 34 kDa, 34 kDa, 26 kDa, and 32 kDa matched with the molecular mass reported previously for *Tr*CE5 (34 kDa) [14] and with the calculated molecular masses based on the protein sequences, which were 33.8 kDa (*An*CE16), 25.8 kDa (*An*CE5), and 32.5 kDa (*Te*CE1). For the commercially available acetyl esterases, protein bands were observed at 37 kDa, 24 kDa, 24 kDa, and 34 kDa, respectively, and these also matched with the calculated molecular masses based on the protein sequences, which were 36.4 kDa (*Ct*CE2), 22.8 kDa (*Ct*CE3), 22.8 kDa (*Ct*CE4), and 34.9 kDa (*Os*CE6).

The purity of the acetyl esterases present in the enzyme solutions was determined by quantitative densitometry, shown in Figure 1 as the proportion (%) of the total proteins present. The SDS-PAGE analysis of the cloned acetyl esterases expressed by *A. niger* ISO527 also showed the presence of protein bands different from those of the cloned enzymes (Figure 1). Particularly for *An*CE5, a range of other protein bands was present, resulting in a lower purity (15%) compared to the other acetyl esterase preparations. Nevertheless, the production of the recombinant enzymes is believed to be functionally specific, since production of native enzymes acting on complex carbohydrates is not induced by the expression and cultivation system used (no further data shown) [15]. An assay to verify the absence of other monosaccharide-releasing side activities was performed. No monosaccharide-releasing activities were apparent after incubation of water unextractable solids (WUS) with the acetyl esterase-containing solutions (*Tr*CE1, *Ct*CE2, *Ct*CE3, *Ct*CE4, *Tr*CE5, *An*CE5, *Os*CE6, and *An*CE16; results not shown). This confirms a selective secretion of the cloned esterases by *A. niger* ISO527, as observed previously [15]. No other acetyl esterases are assumed to be present due to the specific protein expression and the absence of deviating or unexpected effects during long (16-h) incubation times, monitored by NMR analysis, as described below. The absence of interfering side activities and the presence of significant amounts of each acetyl esterase enabled investigation of their modes of action.

### Activity of acetyl esterases

#### Activity on p-nitrophenyl acetate (p-NP-Ac)

**Figure 2:**
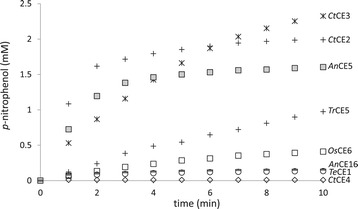
***p*** **-Nitrophenyl acetate assay.** *p*-Nitrophenol released by incubation of *p*-nitrophenyl acetate with acetyl esterases (*Te*CE1, *Ct*CE2, *Ct*CE3, *Ct*CE4, *Tr*CE5, *An*CE5, *Os*CE6, and *An*CE16).

The generic esterase substrate *p*-NP-Ac was used to verify the presence of acetyl esterase activity. The release of *p*-nitrophenol by the hydrolysis of *p*-NP-Ac is shown in Figure 2. The serine-type acetyl esterases, *Te*CE1, *Ct*CE2, *Ct*CE3, *An*CE5, *Tr*CE5, *Os*CE6, and *An*CE16, readily hydrolyzed *p*-NP-Ac. The non-serine-type, metal-dependent esterase *Ct*CE4 was not active on *p*-NP-Ac. The highest *p*-nitrophenol release was observed for the bacterial CE2 and CE3 esterases from *C. thermocellum* (*Ct*CE2 and *Ct*CE3). The CE5 acetyl xylan esterases (*An*CE5 and *Tr*CE5) hydrolyzed *p*-NP-Ac more efficiently than *Te*CE1, *Os*CE6, and *An*CE16. The hydrolysis of *p*-NP-Ac only gives a rough indication of the expected hydrolytic activity on acetylated oligosaccharides. Hence, characterization of the acetyl esterases was continued with partially acetylated plant xylo-oligosaccharides.

#### Deacetylation efficiency of acetylated xylo-oligosaccharides

**Figure 3:**
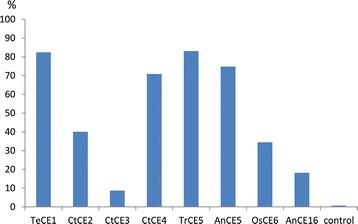
**Acetic acid assay.** Acetic acid released as proportion (%) of the total amount of acetic acid present in acetylated 4-*O*-methyl glucuronic acid (MeGlcA)-substituted xylo-oligosaccharide (AcUXOS) after an incubation time of 16 h, at pH 5.0 and 50°C.

The acetic acid released from AcUXOS after an incubation time of 16 h with acetyl esterases from different families as determined by ^1^H NMR is shown in Figure 3. All acetyl esterases showed deacetylation of AcUXOS. High levels of acetic acid release were obtained with the fungal *Te*CE1, *Tr*CE5, and *An*CE5 and the bacterial *Ct*CE4. The efficient deacetylation by CE5 esterases is in agreement with the reported high deacetylation of poly- and oligosaccharides by Axe1 from *T. longibrachiatum*, which belongs to CE family 5 [10]. *Ct*CE4 efficiently deacetylated AcUXOS, in contrast to its absence of activity towards *p*-NP-Ac (Figure 2). This indicates that activities by acetyl esterases can differ significantly when they act on acetylated oligosaccharides or on generic substrates.

Moderate acetic acid release was obtained for *Ct*CE2, *Os*CE6, and *An*CE16 (Figure 3). Hydrolytic activity by *Ct*CE2 from *Neocallimastix patriciarum* towards acetylated glucuronoxylan has been reported before [16]. *Os*CE6 has been reported to remove acetyl groups from 2-*O*, 3-*O*, and 2,3-di-*O* acetylated Xyl*p* [5,6].

Although the positions of all acetyl groups over the oligomeric substrate AcUXOS are not known, the number of acetyl groups removed by *An*CE16 (18%) matches with the level of reducing end groups present as estimated from the mass spectrum obtained previously [8]. The data suggest exo-acting activity for the CE16 enzyme from *Aspergillus niger*, as proposed previously for the CE16 acetyl esterase from *Trichoderma reesei* [17]. So far, there is no structural information available for the CE16 family [18], indicating a further need for characterization of this CE family. Only low deacetylation was obtained for *Ct*CE3, showing that this enzyme is not efficiently de-esterifying AcUXOS under the hydrolysis conditions applied.

### Structural characterization of AcUXOS

**Figure 4:**
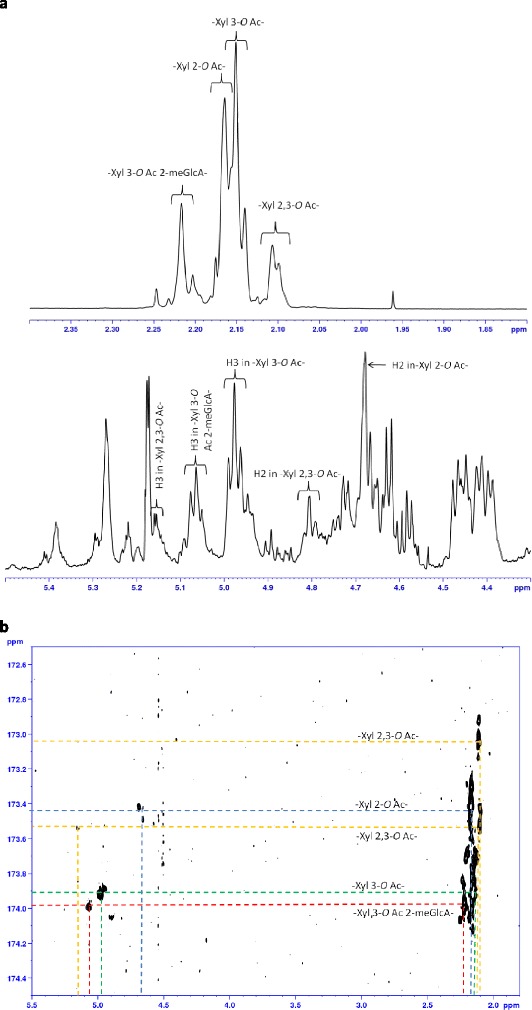
**NMR spectra of acetylated 4-** ***O*** **-methyl glucuronic acid-substituted xylo-oligosaccharides. (a)** ^1^H NMR and **(b)** ^13^C HMBC spectra of acetylated 4-*O*-methyl glucuronic acid (MeGlcA)-substituted xylo-oligosaccharides (AcUXOS) from *E. globulus*. Designations are the same as in Table 1.

The AcUXOS fraction was further used to determine the positional specificity of acetyl esterases. In order to characterize the acetylation pattern of AcUXOS, ^1^H and 2D NMR measurements were performed. The relevant acetylated structural units of the eucalyptus xylo-oligosaccharides were assigned using literature data [19] and confirmed by the reporter peaks observed for the AcUXOS fraction by ^1^H and ^13^C heteronuclear multiple-bond correlation spectroscopy (HMBC) spectra (Table 1, Figure 4). Due to the focus of this study on monitoring the deacetylation of the structural units by various enzymes in time, a full assignment of all cross peaks was not needed. The presence of the targeted structural units was confirmed by reporter peaks (Table 1), which were in agreement with literature data [5,19]. The acetyl positions and their corresponding chemical shifts for the observed 2-*O*, 3-*O*, and 2,3-di-*O* acetylated Xyl*p* and the 3-*O* acetylated α-1,2-MeGlcA-substituted Xyl*p* residues are shown in Table 1. The regions used for the integration of the different acetylated structural units (^1^H NMR and ^13^C HMBC spectra) are shown in Figure 4. The NMR measurement showed that approximately 26% of the acetyl groups present in AcUXOS are attached at *O*-2 and 46% at *O*-3 of mono-acetylated Xyl*p*, 15% represent di-acetylated Xyl*p*, and 13% are attached at *O*-3 of α-1,2-MeGlcA-substituted Xyl*p*. Discrimination between acetyl groups present on “internal” Xyl*p* or on Xyl*p* at the reducing and non-reducing end was not made. On the ^1^H NMR spectra, the signals corresponding to the acetyl groups present at the reducing and non-reducing end and on “internal” Xyl*p* overlap. The ^1^H NMR spectra obtained (Figure 4) showed separated resonances (2.10/2.11, 2.15, 2.17, and 2.22 ppm) for the different, acetylated structural units (−Xyl 2,3-*O* Ac-, −Xyl 3-*O* Ac-, −Xyl 2-*O* Ac-, and -Xyl 3-*O* Ac 2-MeGlcA-) present in AcUXOS (Table 1). By monitoring the changes in signal intensities for the region 2.1 to 2.22 ppm during incubation with acetyl esterases, the deacetylation efficiency and preference towards differently acetylated Xyl*p* can be investigated.

**Table 1:** **Chemical shifts of acetylated Xyl** ***p***

**Structural unit**	**H2**	**H3**	**COC** ***H***_***3***_	**C2**	**C3**	***C*** **OCH**_**3**_
-Xyl2-*O*Ac-	4.68	-	2.17	73.39	-	173.42
-Xyl3-*O*Ac-	-	4.98	2.15	-	75.18	173.92
-Xyl2,3-*O*Ac-	4.81	5.15	2.10/2.11	71.30	72.78	173.04/173.53
-Xyl3-*O*Ac2-MeGlcA-	-	5.06	2.22	-	73.77	173.99

Assignment of relevant chemical shifts of acetyl residues present in 4-*O*-methyl glucuronic acid (MeGlcA)-substituted xylo-oligosaccharides (AcUXOS) from *E. globulus*.

### Positional specificity

**Figure 5:**
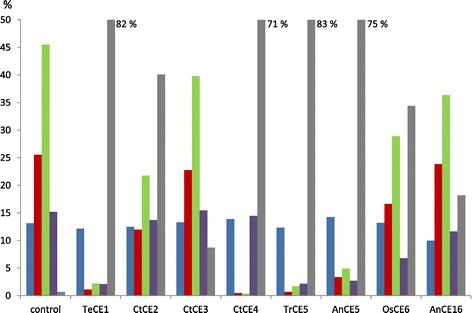
**Deacetylation by acetyl esterases from different carbohydrate esterase families.** Distribution of acetyl moieties over 2-*O*, 3-*O*, and 2,3-di-*O* acetylated Xyl*p*, and 3-*O* acetylated Xyl*p* α-1,2 substituted with MeGlcA present in acetylated 4-*O*-methyl glucuronic acid (MeGlcA)-substituted xylo-oligosaccharides (AcUXOS) from *E. globulus* and free acetic acid as proportion (%) of the sum of the total acetyl groups present after incubation of AcUXOS with acetyl esterases from different CE families (incubation time: 16 h). Structural units: (blue) -Xyl 3-*O* Ac 2-MeGlcA-, (red) -Xyl 2-*O* Ac-, (green) -Xyl 3-*O* Ac-, (purple) -Xyl 2,3-*O* Ac-, (gray) acetic acid; the control represents AcUXOS without enzyme treatment.

The distribution of acetyl moieties over 2-*O*, 3-*O*, and 2,3-di-*O* acetylated Xyl*p* and 3-*O* acetylated Xyl*p* 2-O substituted with MeGlcA and the free acetic acid obtained after an incubation of 16 h (endpoint) of AcUXOS with acetyl esterases from different families is shown in Figure 5. This distribution was obtained by monitoring the changes in signal intensities for the region 2.1 to 2.22 ppm, corresponding to the structural units (Figure 4). The acetyl esterases can be divided in three groups with respect to their positional specificity. The three groups showed activity towards 2-*O* and 3-*O* acetylated Xyl*p* or towards 2-*O*, 3-*O*, and 2,3-di-*O* acetylated Xyl*p* or towards 2-*O*, 3-*O*, and 2,3-di-*O* acetylated Xyl*p* and 3-*O* acetylated Xyl*p* 2-*O* substituted with MeGlcA. The deacetylation of 2,3-di-*O* acetylated Xyl*p* generates 2-*O* and 3-*O* acetylated Xyl*p* moieties. Even though doubly acetylated Xyl*p* represents a relatively small fraction of the total acetylation (15%), the deacetylation efficiency determined must be interpreted in the context of small amounts of 2-*O* and 3-*O* acetylated Xyl*p* being generated if the enzyme is active on diacetylated Xyl*p*. In the case of *Te*CE1, *Tr*CE5, and *An*CE5, 2-*O*, 3-*O*, and 2,3-di-*O* acetylated Xyl*p* were nearly completely deacetylated, as discussed below. Therefore, the generation of 2-*O* and 3-*O* acetylated Xyl*p* from 2,3-di-*O* acetylated Xyl*p* is not relevant regarding the deacetylation efficiencies obtained for these enzymes. However, for *Os*CE6 and *An*CE16, the deacetylation efficiency towards 2-*O* and 3-*O* acetylated Xyl*p* may be slightly underestimated, as small amounts of 2-*O* and 3-*O* acetylated Xyl*p* were generated. Monitoring of the initial 30 min of the hydrolysis, as discussed below, allowed a clear determination of the activity of esterases towards different acetylated Xyl*p*.

#### Acetyl esterases group I: activity exclusively towards 2-O and 3-O acetylated Xylp

The bacterial acetyl esterases from *C. thermocellum* (*Ct*CE2, *Ct*CE3, and *Ct*CE4) showed deacetylation of 2-*O* and 3-*O* acetylated Xyl*p*. No activity was observed towards 2,3-di-*O* acetylated Xyl*p* and 3-*O* acetylated Xyl*p* 2-*O* substituted with MeGlcA. Complete deacetylation of 2-*O* and 3-*O* acetylated Xyl*p* was obtained for *Ct*CE4. Hydrolysis of the acetyl groups at these positions has also been described for the CE4 acetyl esterase from *S. lividans* on aspen oligosaccharides [5,6]. The activity of the enzyme was shown to be metal ion-dependent and the enzyme was able to deacetylate positions 2 and 3 from the synthetic substrate methyl β-D-xylopyranoside [20,21]. As the ^1^H signals show complete deacetylation of 2-*O* and 3-*O* acetylated Xyl*p*, activity of *Ct*CE4 towards terminal and internal acetylated xylopyranosyl units can be concluded. The presence of terminal acetylated xylo-oligosaccharides in AcUXOS has previously been shown by mass spectrometry [8,10]. Apparently, activity towards 2-*O* and 3-*O* acetylated Xyl*p* was not hindered by substituents (such as MeGlcA) that could be present on neighboring xylopyranosyl units. A moderate deacetylation efficiency of these positions (52% of both the 2-*O* and 3-*O* acetylated Xyl*p*, were deacetylated) was observed for *Ct*CE2. CE2-classified esterases were described to act as 6-*O*-deacetylases, deacetylating the 6 position of hexopyranosyl residues [22]. Therefore, the activity towards acetylated xylan might only be a fraction of the activity towards other acetylated types of polysaccharides [3]. *Ct*CE2 from *C. thermocellum* has a somewhat unique position within the CE2 family, as the active site acts as an esterase and cellulose binding domain, being an example of “gene sharing” [23]. Therefore, further studies on the deacetylation activity of other representatives of the CE2 family are required in order to determine whether this dual activity might affect the deacetylation efficiency and specificity compared to other members of this family. Activity on the synthetic substrates (4-*O*-acetyl 4-nitrophenyl β-D-xylopyranoside, methyl 3,4-*O*-diacetyl- and methyl 2,4-*O*-diacetyl-β-D-xylopyranoside) has been reported for *Ct*CE2, suggesting a preference towards the 4-*O* position, which was also observed for the CE2 esterase from *Cellvibrio japonicus* [22]. However, in AcUXOS, the 4-*O* position is not acetylated. The data show that in the absence of the 4-O acetyl group, a clear decrease of the 2-*O* and 3-*O* acetylated Xyl*p*, representing approximately 40% of the total acetylation present in AcUXOS, was obtained with *Ct*CE2. Figure 3 shows that *Ct*CE3 was also able to hydrolyze both 2-*O* and 3-*O* acetylated Xyl*p*, although the deacetylation efficiency was low under the conditions applied. Approximately 9% of the total acetylation present in AcUXOS was removed. The activity by *Ct*CE3 towards acetylated xylo-oligosaccharides has previously been shown by its activity towards birchwood xylan [24].

#### Acetyl esterases group II: activity towards 2-O, 3-O, and 2,3-di-O acetylated Xylp

Clearly decreased ^1^H signals for 2-*O*, 3-*O*, and 2,3-di-*O* acetylated Xyl*p* were obtained after incubation of AcUXOS with *Te*CE1, *Tr*CE5, *An*CE5, and *Os*CE6, showing deacetylation of Xyl*p* at these positions. In contrast, no activity towards the 3-*O* acetylated Xyl*p* 2-*O* substituted with MeGlcA was observed.

For *Te*CE1, *Tr*CE5, and *An*CE5 the deacetylation at the 2-*O*, 3-*O*, and 2,3-di-*O* positions was nearly complete. As described above, complete deacetylation implies activity towards terminal and internal acetylated xylopyranosyl and also tolerance towards MeGlcA substituents that might be present on neighboring xylopyranosyl units. These three enzymes are efficiently deacetylating AcUXOS, releasing up to 83% of the acetyl groups present in AcUXOS, with only the 3-*O* acetylated Xyl*p* 2-*O* substituted with MeGlcA (which comprises 13% of the total acetyl residues initially present) being recalcitrant towards hydrolysis.

#### Acetyl esterases group III: activity towards 2-O, 3-O, and 2,3-di-O acetylated Xylp and 3-O acetylated Xylp 2-O substituted with MeGlcA

For *An*CE16, moderate deacetylation of AcUXOS was observed. However, the positional tolerance of this enzyme was the highest of the enzymes tested, as the signals corresponding to 2-*O*, 3-*O*, and 2,3-di-*O* acetylated Xyl*p* and 3-*O* acetylated Xyl*p* 2-*O* substituted with MeGlcA decreased during the hydrolysis. The decrease found for 2-*O* acetylated Xyl*p* after an incubation time of 16 h was low (13%), indicating that *An*CE16 does not hydrolyze acetyl groups efficiently at this position. A low deacetylation activity of 2-*O* acetylated Xyl*p* by the CE16 acetyl esterase from *T. reesei* was also observed on an artificial substrate [9]. Significantly, our data not only show a clear signal decrease for 3-*O* and 2,3-di-*O* acetylated Xyl*p,* but also for the 3-*O* acetylated Xyl*p* 2-*O* substituted with MeGlcA. This is relevant, as previous studies using artificial substrates for the characterization of CE16 acetyl esterases did not include acetylated Xyl*p* carrying a MeGlcA substituent on the same residue [9]. If the deacetylation by *An*CE16 proceeds primarily at the non-reducing end, this would explain the moderate deacetylation efficiency towards acetylated Xyl*p*. This strengthens the hypothesis of a predominant exo-activity by CE16 acetyl esterases from *A. niger* (this study) and *T. reesei* [17]. The NMR data showed no clear positional specificity towards the acetylated positions, while no obvious decrease was observed for the signal corresponding to 2-*O* acetylated Xyl*p*.

#### Deacetylation rates for different acetylated xylopyranosyl units

**Figure 6:**
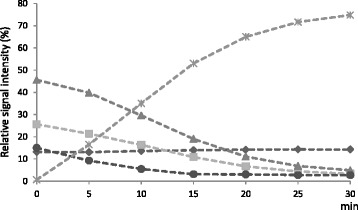
**Deacetylation by acetyl xylan esterase from carbohydrate esterase family 5.** Relative signal intensities (% area of the total area of acetyl groups and acetic acid present) for acetyl xylan esterase CE5 (*A. niger*)-treated acetylated xylo-oligosaccharides. 3-*O* (triangles), 2-*O* (squares), 2,3-di-*O* (circles) acetylated Xyl*p*, 3-*O* acetylated Xyl*p* 2-*O* substituted with MeGlcA (diamonds), and acetic acid released (asterisks).

Endpoint values for the deacetylation of AcUXOS indicate the ability of the acetyl esterases to catalyze the hydrolysis of acetyl groups at a specific location, but do not reflect the positional preferences at the start of hydrolysis. In order to determine the positional preferences on xylo-oligosaccharides, the deacetylation rate of acetyl esterases from different CE families was measured for 2-*O*, 3-*O*, and 2,3-di-*O* acetylated Xyl*p* and 3-*O* acetylated Xyl*p* 2-*O* substituted with MeGlcA after short incubations (5 or 30 min; Table 2). The effect of small amounts of 2-*O* and 3-*O* acetylated Xyl*p* being generated by acetyl esterases with activity towards 2,3-di-*O* acetylated Xyl*p* was discussed above. However, this does not affect the initial deacetylation rates, as all acetylated Xyl*p* are present in excess at the start. Figure 6 shows an example of the hydrolysis curves obtained for the various acetyl positions by incubation with the CE5 acetyl xylan esterase (*An*CE5) from *A. niger* for 30 min. From these hydrolysis curves, the deacetylation rates for each acetylated position (acetyl groups removed/min/mg enzyme, at pH 5.0 and 50°C) were calculated based on the decrease of the resonance corresponding to 2-*O*, 3-*O*, and 2,3-di-*O* acetylated Xyl*p* and 3-*O* acetylated Xyl*p* 2-*O* substituted with MeGlcA. Enzyme-recalcitrant acetyl groups were identified by the presence of a constant signal over the time period, as observed for 3-*O* acetylated Xyl*p* 2-*O* substituted with MeGlcA, in the hydrolysis curve of the sample incubated with *An*CE5 (Figure 6). An additional file shows the hydrolysis curves obtained by incubation with acetyl esterases from different CE families in more detail (see Additional file 1).

**Table 2:** **Deacetylation rates and rates for acetic acid released**

**enzyme**	**-Xyl3-** ***O*** **Ac2-MeGlcA-**	**-Xyl2-** ***O*** **Ac-**	**-Xyl3-** ***O*** **Ac-**	**-Xyl2,3-** ***O*** **Ac-**	**Acetic acid**
*Te*CE1^a^	0.0	12.7	25.5	44.0	74.6
*Ct*CE2^b^	0.0	<0.01	6.5	0.0	5.7
*Ct*CE3^b^	0.0	<0.01	<0.01	0.0	<0.01
*Ct*CE4^b^	0.0	88.1	356.6	0.0	365.7
*An*CE5^a^	0.0	202.7	276.2	274.4	754.7
*Tr*CE5^a^	0.0	171.8	289.2	92.5	540.9
*Os*CE6^a^	0.0	48.64	92.2	50.3	117.7
*An*CE16^b^	0.03	1.80	1.92	1.50	5.25

Deacetylation rates (μmol acetyl groups removed/min/mg enzyme) by acetyl esterases for 2-*O*, 3-*O*, and 2,3-di-*O* acetylated Xyl*p* and 3-*O* acetylated Xyl*p* 2-*O* substituted with MeGlcA from eucalyptus and rates for the total acetic acid released (μmol acetic acid released/min/mg enzyme).

^a^μmol acetyl groups removed/min/mg enzyme and acetic acid released/min/mg enzyme after 5 min.

^b^μmol acetyl groups removed/min/mg enzyme and acetic acid released/min/mg enzyme after 30 min.

Table 2 shows the rates of hydrolysis for the positions investigated. The values obtained (single point measurements) are a guide to their true specific activity. High deacetylation rates were observed for 2-*O* and 3-*O* acetylated Xyl*p* residues by *An*CE5, *Tr*CE5, and *Ct*CE4. Moreover, *An*CE5 and *Tr*CE5 also showed high rates for the hydrolysis of the diacetylated xylopyranosyl units. Lower deacetylation rates were observed for *Te*CE1 and *Os*CE6 compared to *An*CE5 or *Tr*CE5. Finally, low rates were obtained for the bacterial *Ct*CE2 and *Ct*CE3, and these enzymes were not active on diacetylated Xyl*p*. In the case of *Ct*CE3, activity on 2-*O* and 3-*O* acetylated Xyl*p* could only be concluded from the overnight incubation (Figure 5).

Regarding *An*CE16, a relatively low activity towards 2-*O*, 3-*O*, and 2,3-di-*O* acetylated Xyl*p* and 3-*O* acetylated Xyl*p* 2-*O* substituted with MeGlcA was observed from monitoring the first 30 min of the hydrolysis (Table 2). Also in this case, hydrolysis at these positions was clearly observed with the overnight incubation (Figure 5).

#### Positional preference on acetylated xylo-oligosaccharides

In order to evaluate whether acetyl esterases showed a preference for certain positions, the ratio of the deacetylation rate and the relative abundance of the acetyl groups present at a specific position was calculated (Table 3). For some enzymes (*Ct*CE2, *Ct*CE3, and *An*CE16), the deacetylation activity was too low to determine clear positional preferences.

**Table 3:** **Positional preferences of acetyl xylan esterases**

**Enzyme**	**-Xyl3-** ***O*** **Ac2-MeGlcA-**	**-Xyl2-** ***O*** **Ac-**	**-Xyl3-** ***O*** **Ac-**	**-Xyl2,3-** ***O*** **Ac-**
*Te*CE1	0	12	14	74
*Ct*CE2	0	n.d.	n.d.	0
*Ct*CE3	0	n.d.	n.d.	0
*Ct*CE4	0	30	70	0
*An*CE5	0	24	19	57
*Tr*CE5	0	35	33	32
*Os*CE6	0	26	28	46
*An*CE16	n.d.	n.d.	n.d.	n.d.

Positional preference on acetylated xylo-oligosaccharides, calculated as the positional rate per relative amount of acetyl groups present (2-*O*, 3-*O*, 2,3-di-*O* acetylated Xyl*p* and 3-*O* acetylated Xyl*p* 2-*O* substituted with MeGlcA) at the start of hydrolysis. The data were normalized.

0, no activity; n.d., not determined.

The data in Table 3 indicate a clear preference towards doubly acetylated Xyl*p* for *Te*CE1. A preference towards diacetylated xylopyranosyl units has also been described for the CE1 acetyl xylan esterase from *S. commune* [6]. For *Ct*CE4, a preference for the 3-*O* acetylated Xyl*p* was observed, with a ratio more than twice as high as that for the 2-*O* acetylated Xyl*p. An*CE5 and *Os*CE6 showed a preference for 2,3-di-*O* acetylated Xyl*p* over 2-*O* and 3-*O* acetylated Xyl*p*. No such positional preference was observed for *Tr*CE5, which deacetylated all positions with similar preference (except 3-*O* acetylated Xyl*p* 2-*O* substituted with MeGlcA). The difference in preference of the CE5 esterases *Tr*CE5 and *An*CE5 shows that acetyl xylan esterases belonging to the same CE family but different species do not necessarily have the same positional preference. Consequently, sequence-based classification of esterases cannot fully predict the positional preferences of these enzymes. Multiple representatives of one enzyme class will need to be compared in order to conclude whether positional preferences are conserved for different CE families.

The aim of this study was to determine the specificity (positional preference) of a number of acetyl esterases from different CE families. As the main indicator for this we used the rates of hydrolysis shown in Table 2. To allow the use of this information in industrial practice, incubation conditions were used that may be considered relevant for biofuel production [12,13]. These conditions do not necessarily reflect the pH and temperature optima of a particular esterase. It is clear that this would affect the absolute activity measured. It is less clear whether this could also affect the positional preference, and this limitation must be considered when using the preferences reported here.

## Conclusions

Depending on the acetylation pattern of a particular substrate, suitable acetyl esterases may be selected from these data (Tables 2 and 3). Knowledge of the deacetylation efficiency and the positional preference of fungal and bacterial acetyl esterases allows the design of optimized enzyme mixtures. The acetyl esterases tested could be categorized into three groups with activities towards (i) 2-*O* and 3-*O* and (ii) 2-*O*, 3-*O*, and 2,3-di-*O* acetylated Xyl*p*, and (iii) 2-*O*, 3-*O*, and 2,3-di-*O* acetylated Xyl*p* and 3-*O* acetylated Xyl*p* 2-*O* substituted with MeGlcA. High deacetylation rates were obtained for *Ct*CE4, *Tr*CE5, and *An*CE5 (Table 2) under the conditions employed. Positional preferences towards 2,3-di-*O* acetylated Xyl*p* (*Te*CE1, *An*CE5, and *Os*CE6) or 3-*O* acetylated Xyl*p* (*Ct*CE4) were found. For the deacetylation of acetylated 4-*O*-methyl glucuronic acid-substituted xylo-oligosaccharides a combination of the CE5 (*Tr*CE5 or *An*CE5) and the CE16 acetyl esterases (*An*CE16) would seem to be the most efficient.

## Methods

### Substrates

*Eucalyptus globulus* xylan hydrolysate was kindly donated by Dr. J.C. Parajo of the University of Vigo-Ourense, Spain [25]. The acetylated 4-*O*-methyl glucuronic acid (MeGlcA)-substituted xylo-oligosaccharide (AcUXOS) fraction of the xylan hydrolysate was described previously [8]. The Xyl, Ara, Gal, and MeGlcA contents of AcUXOS were 765, 0, 42, and 207 mg g^−1^ dry matter (DM) [8]. The acetic acid was measured by ^1^H NMR, and was 210 mg g^−1^ DM. Corn silage water unextractable solids (WUS) were used and have been described [1].

### Enzymes

Cloning and expression of the acetyl esterase 1 from *Trichoderma reesei* belonging to CE family 5 (*Tr*CE5, *T. reesei*, CAA93247.1) has been described previously [15]. Acetyl esterases from *Aspergillus niger* belonging to CE families 5 and 16 [*An*CE5 (CAK49022.1), *An*CE16 (CAK45102.1)] and the CE1 classified acetyl esterase from *Talaromyces emersonii* [*Te*CE1 (ADX07526.1)] were obtained from DSM (Heerlen, The Netherlands). Expression of these genes in *A. niger* ISO527 was performed according to the procedure described previously [15]. Acetyl esterases classified as belonging to CE families 2 [*Ct*CE2 (AAA23224)], 3 [*Ct*CE3 (ABN52033)], and 4 [*Ct*CE4 (ABN54169)] from *Clostridium thermocellum* were purchased from Prozomix (Haltwhistle, UK). The CE6 classified acetyl esterase [*Os*CE6 (AAC14690.1)] from *Orpinomyces* sp*.* PC-2 was purchased from Megazyme (Wicklow, Ireland).

### Electrophoresis

SDS-PAGE was performed with a NuPAGE 10% Bis-Tris gel (Life Technologies, Carlsbad, CA, USA) and a Power Ease 500 system (Life Technologies). The protein bands were stained with the Instant Blue protein gel stain (Expedeon Inc., San Diego, CA, USA). The protein markers Mark 12 and SeeBlue Plus 2 (Life Technologies) were used. Quantification of the protein bands obtained by densitometry was performed with the software ImageJ, developed at the National Institute of Health (Bethesda, MD, USA).

### Calculation of molecular masses of proteins

The protein sequences of the acetyl esterases [*Te*CE1, *An*CE5, *Tr*CE5, and *An*CE16] were retrieved from the National Center for Biotechnology Information (Bethesda, MD, USA, www.ncbi.nlm.nih.gov) and calculated with the Compute pI/Mw tool (ExPASy Bioinformatics Resource Portal, www.expasy.org).

### *p*-Nitrophenyl acetate (*p*-NP-Ac) assay

Enzyme assays with the acetyl esterases using *p*-NP-Ac were performed as described previously [15]. The enzymes were applied at a dosage of 0.3 mg total protein mL^−1^ in sodium acetate buffer (10 mM, pH 5.0) at 40°C. The *p*-nitrophenol release was determined by continuous measurement for 10 min at 405 nm.

### Monosaccharide-releasing activities assay

WUS (10 g L^−1^) was incubated with acetyl xylan esterases (10 g protein kg^−1^ substrate) in sodium citrate buffer (10 mM, pH 5.0, containing sodium azide 0.5 g kg^−1^) at 50°C and 700 rpm for 8 h. The enzymes were heat inactivated (10 min, 98°C) and analyzed for the presence of monosaccharides (mg monosaccharides released g^−1^ dry matter) on an HPAEC system as described previously [1].

### NMR

^*1*^*H and 2D NMR:*^1^H-^13^C HSQC spectra were recorded on an Avance III 700 MHz spectrometer (Bruker BioSpin, Billerica, MA, USA), equipped with a helium-cooled cryoprobe. The HSQC spectra were recorded using a correlation via double inept transfer pulse program using sensitivity improvement (hsqcetgpsi2), in 32 scans, with 32 dummy scans, 512 increments, sweep width 130 ppm, relaxation delay 1.2 s, and acquisition time 0.36 s.

^1^H-^13^C HMBC spectra were recorded using heteronuclear zero and double quantum coherence with a twofold low-pass J-filter constant time version (shmbcctetgpl2nd), in 64 scans, with 16 dummy scans, 1024 increments, sweep width 20 ppm, relaxation delay 1.2 s, and acquisition time 0.24 s.

### NMR enzyme assay

AcUXOS was dissolved in deuterated sodium citrate buffer (10 mM, pH 5.0) at a concentration of 5 g L^−1^, transferred to NMR tubes (3 x 103.5 mm glass tubes) and heated to 50°C. A ^1^H NMR spectrum of each sample was measured before the addition of enzymes. A concentration of 5 g kg^−1^ substrate for the acetyl esterases *Te*CE1, *Ct*CE2, *Ct*CE3, *Ct*CE4, *Tr*CE5, *An*CE5, *Os*CE6, and *An*CE16 was determined as suitable for the purpose of measurement. *Ct*CE4 was also dosed at 0.1 g kg^−1^ substrate, as this enzyme showed a high hydrolytic rate (data not shown). The samples were vortex mixed after addition of the acetyl esterases. The incubations were done at 50°C and monitored by continuous measurement of the ^1^H NMR spectra for 30 min and a single measurement after 16 h on an Avance III 700 MHz spectrometer (Bruker).

### Calculation of deacetylation rates

The concentration of acetic acid present in the hydrolysate was calculated from the ^1^H NMR spectrum as described previously [26] using the integral area of the internal standard 4,4-dimethyl-4-silapentane-1-sulfonic acid. Concentrations of 2-*O*, 3-*O*, and 2,3-di-*O* acetylated Xyl*p* residues and 3-*O* acetylated Xyl*p* residues 2-*O* substituted with MeGlcA were calculated accordingly based on the resonances obtained by ^1^H NMR analysis; the values were 2.17, 2.15, 2.11/2.10, and 2.22 ppm, respectively. The rates of the acetic acid release and the deacetylation rates for the different locations of acetyl within the Xyl*p* residues (2-*O*, 3-*O*, 2,3-di-*O* acetylated Xyl*p*, and the 3-*O* acetylated Xyl*p* 2-*O* substituted with MeGlcA) were calculated as μmol acetic acid released/min/mg enzyme and μmol specific acetyl group removed/min/mg enzyme, respectively, after a measurement time of 5 min for the relatively fast acetyl esterases (TeCE1, TrCE5, *An*CE5, and *Os*CE6), dosed at 5 g protein kg^−1^ substrate, and after 30 min for the relatively slow acetyl esterases (*Ct*CE2, *Ct*CE3, and *An*CE16), dosed at 5 g protein kg^−1^ substrate, and for the *Ct*CE4, dosed at 0.1 g protein kg^−1^ substrate. The purity of acetyl esterases present in the enzyme solutions was determined by quantitative densitometry of the gels obtained by the SDS-PAGE analysis, as described above. The hydrolytic rates were calculated per actual mg acetyl esterase present.

## Additional file

Additional file 1:
**Positional preferences of acetyl esterases from different CE families towards 4-** ***O*** **-methyl glucuronic acid-substituted xylo-oligosaccharides.** Relative signal intensities (% area of the total area of acetyl groups and acetic acid present) for enzyme treated acetylated xylo-oligosaccharides with acetyl esterase from different CE families. 3-*O* (triangles), 2-*O* (squares), 2,3-di-*O* (circles), acetylated Xyl*p*, 3-*O* acetylated Xyl*p* 2-*O* substituted with MeGlcA (diamonds), and acetic acid released (asterisks).

## Abbreviations

AcUXOS: acetylated 4-*O*-methyl glucuronic acid-substituted xylo-oligosaccharides
*An*CE5: CE5 classified acetyl esterase from *Aspergillus niger*
*An*CE16: CE16 classified acetyl esterase from *Aspergillus niger*
CtCE2: CE2 classified acetyl esterase from *Clostridium thermocellum*
*Ct*CE3: CE3 classified acetyl esterase from *Clostridium thermocellum*
*Ct*CE4: CE4 classified acetyl esterase from *Clostridium thermocellum*
DM: dry matter
HMBC spectroscopy: heteronuclear multiple-bond correlation spectroscopy
HPAEC: high-performance anion exchange chromatography
HSQC spectroscopy: heteronuclear single quantum coherence spectroscopy
MeGlcA: methyl glucuronic acid
NMR: nuclear magnetic resonance
*Os*CE6: CE6 classified acetyl esterase from *Orpinomyces* sp*.* PC-2
*p*-NP-Ac: *p*-nitrophenyl acetate
SDS-PAGE: sodium dodecyl sulfate polyacrylamide gel electrophoresis
*Te*CE1: CE1 classified acetyl esterase from *Talaromyces emersonii*
*Tr*CE5: CE5 classified acetyl esterase from *Trichoderma reesei*
WUS: water unextractable solids
Xyl*p*: xylopyranosyl unit
