# Supplementary material for: Positional preferences of acetyl esterases from different CE families towards acetylated 4-O-methyl glucuronic acid-substituted xylo-oligosaccharides
Source: Biotechnol Biofuels. 2015 Jan 22;8:7. doi: 10.1186/s13068-014-0187-6 (PMC4311478; doi:10.1186/s13068-014-0187-6)
Supplement: Additional file 1: — Positional preferences of acetyl esterases from different CE families towards 4- O -methyl glucuronic acid-substituted xylo-oligosaccharides. Relative signal intensities (% area of the total area of acetyl groups and acetic acid present) for enzyme treated acetylated xylo-oligosaccharides with acetyl esterase from different CE families. 3-O (triangles), 2-O (squares), 2,3-di-O (circles), acetylated Xylp, 3-O acetylated Xylp 2-O substituted with MeGlcA (diamonds), and acetic acid released (asterisks). [file 13068_2014_187_MOESM1_ESM.docx]

## Additional file 1 – Positional preferences of acetyl esterases from different CE families towards 4-*O*-methyl-glucuronic acid substituted xylo-oligosaccharides

| *Te*CE1  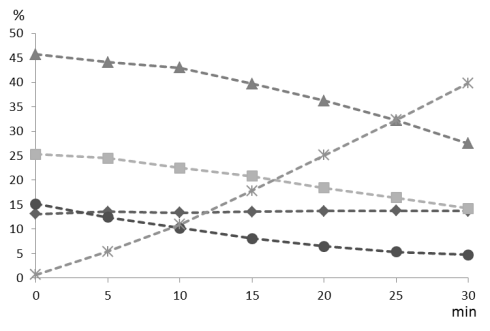 | *Ct*CE2  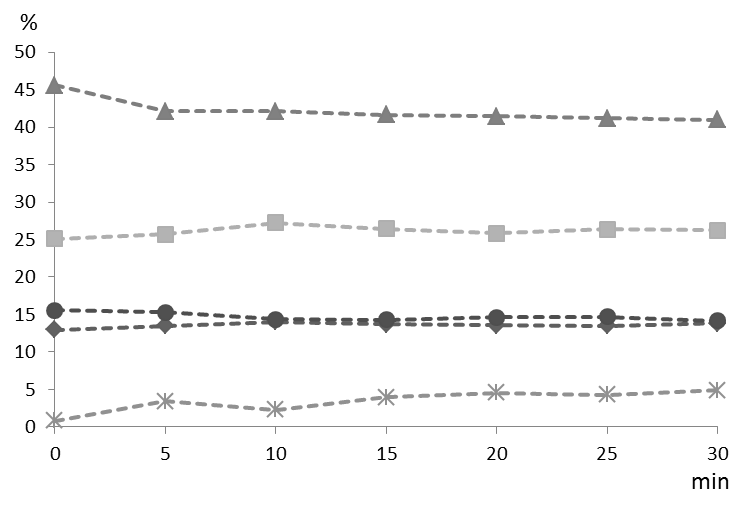 |
| --- | --- |
| *Ct*CE3  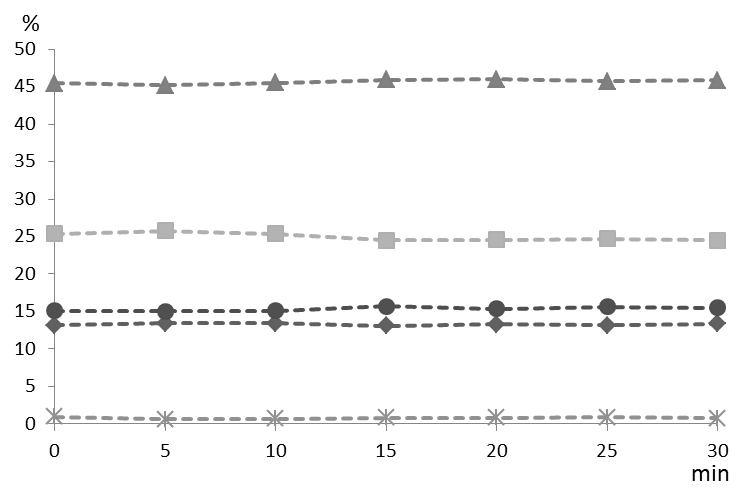 | *Ct*CE4  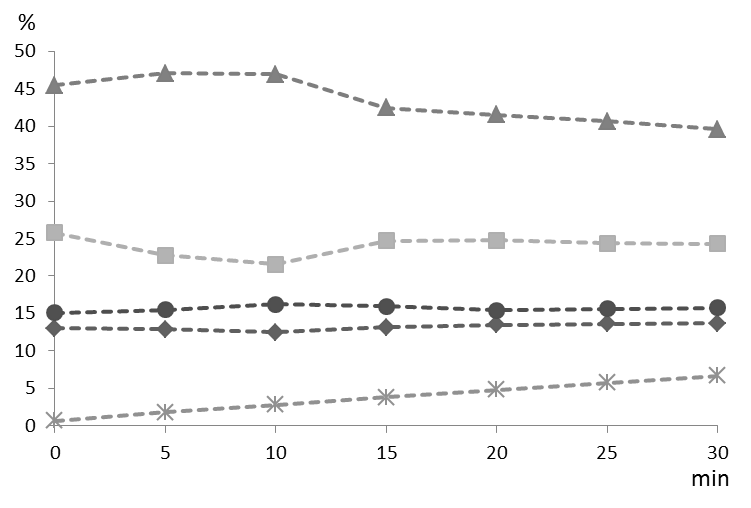 |
| *Tr*CE5  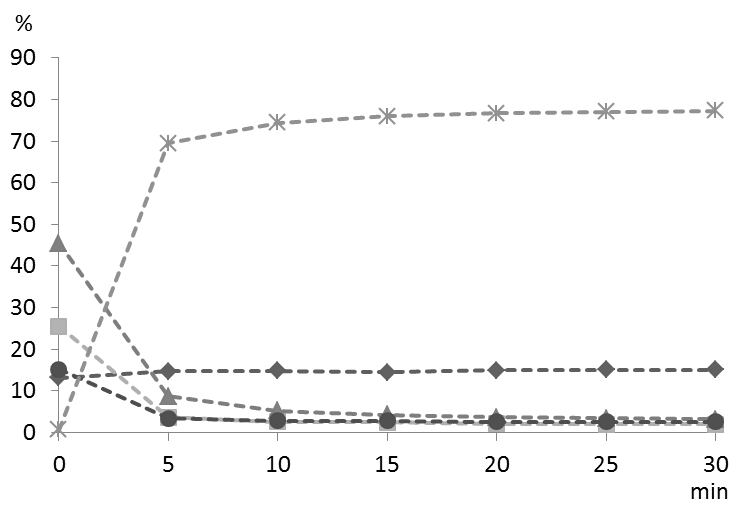 | *An*CE5  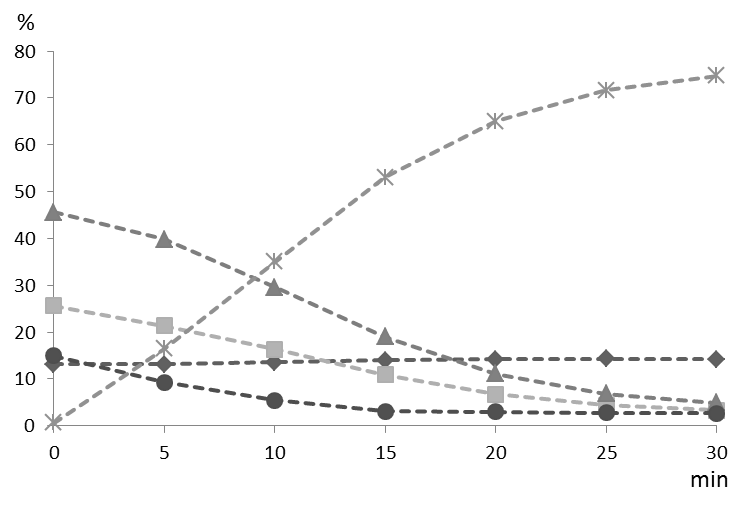 |
| *Os*CE6  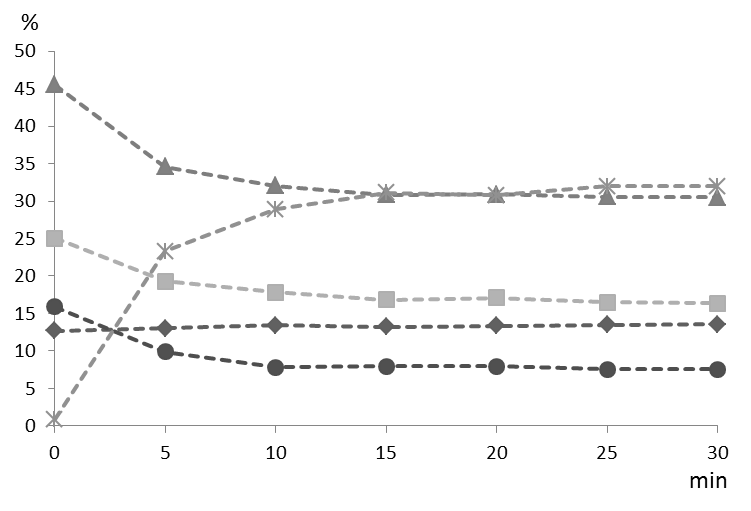 | *An*CE16  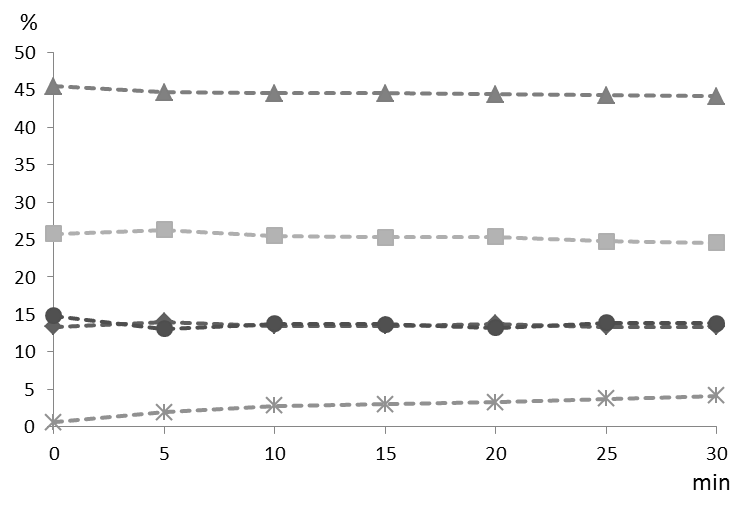 |
